# Morbidity from Malaria in Children in the Year after They Had Received Intermittent Preventive Treatment of Malaria: A Randomised Trial

**DOI:** 10.1371/journal.pone.0023391

**Published:** 2011-08-12

**Authors:** Amadou T. Konaté, Jean Baptiste Yaro, Amidou Z. Ouédraogo, Amidou Diarra, Adama Gansané, Issiaka Soulama, David T. Kangoyé, Youssouf Kaboré, Espérance Ouédraogo, Alphonse Ouédraogo, Alfred B. Tiono, Issa N. Ouédraogo, Daniel Chandramohan, Simon Cousens, Paul J. Milligan, Sodiomon B. Sirima, Brian M. Greenwood, Diadier A. Diallo

**Affiliations:** 1 Centre National de Recherche et de Formation sur le Paludisme (CNRFP), Ouagadougou, Burkina Faso; 2 Department of Disease Control, London School of Hygiene and Tropical Medicine (LSHTM), London, United Kingdom; 3 Department of Infectious Disease Epidemiology, London School of Hygiene and Tropical Medicine, London, United Kingdom; Burnet Institute, Australia

## Abstract

**Background:**

Interventions that reduce exposure to malaria infection may lead to delayed malaria morbidity and mortality. We investigated whether intermittent preventive treatment of malaria in children (IPTc) was associated with an increase in the incidence of malaria after cessation of the intervention.

**Methods:**

An individually randomised, trial of IPTc, comparing three courses of sulphadoxine pyrimethamine (SP) plus amodiaquine (AQ) with placebos was implemented in children aged 3–59 months during the 2008 malaria transmission season in Burkina Faso. All children in the trial were given a long lasting insecticide treated net; 1509 children received SP+AQ and 1505 received placebos. Passive surveillance for malaria was maintained until the end of the subsequent malaria transmission season in 2009, and active surveillance for malaria infection, anaemia and malnutrition was conducted.

**Results:**

On thousand, four hundred and sixteen children (93.8%) and 1399 children (93.0%) initially enrolled in the intervention and control arms of the trial respectively were followed during the 2009 malaria transmission season. During the period July 2009 to November 2009, incidence rates of clinical malaria were 3.84 (95%CI; 3.67–4.02) and 3.45 (95%CI; 3.29–3.62) episodes per child during the follow up period in children who had previously received IPT or placebos, indicating a small increase in risk for children in the former intervention arm (IRR = 1.12; 95%CI 1.04–1.20) (P = 0.003). Children who had received SP+AQ had a lower prevalence of malaria infection (adjusted PR: 0.88 95%CI: 0.79–0.98) (P = 0.04) but they had a higher parasite density (P = 0.001) if they were infected. There was no evidence that the risks of moderately severe anaemia (Hb<8 g/dL), wasting, stunting, or of being underweight in children differed between treatment arms.

**Conclusion:**

IPT with SP+AQ was associated with a small increase in the incidence of clinical malaria in the subsequent malaria transmission season.

**Trial Registration:**

ClinicalTrials.gov NCT00738946

## Introduction

Intermittent preventive treatment (IPT) of malaria is a promising strategy for malaria control in infants and young children. Meta analysis indicates a 30% (95%CI: 20-39) protective efficacy in infants against clinical malaria [1]. Studies conducted in older children living in areas where malaria transmission is highly seasonal have reported 67% to 86% reductions in clinical malaria [2]–[4]. These initial trials were conducted in communities where the use of insecticide treated bednets (ITNs) was low. More recently, trials of IPT in children who were sleeping under a long lasting insecticide treated net (LLIN) showed a protective efficacy against clinical episodes of malaria of 70% in Burkina Faso [5] and 83% Mali [6], indicating that IPT provides substantial additional protection to that offered by ITNs.

Interventions which reduce exposure to malaria could interfere with the development of acquired immunity which could, in turn, lead to a shift of morbidity and mortality from malaria from younger to older age groups (“rebound effect”) [7]. Early studies in African children which employed chemoprophylaxis showed a significant increase in cases of clinical malaria when the intervention was stopped. In The Gambia, children who had received seasonal chemoprophylaxis from the age of three months to five years had a significant increase in clinical episodes of malaria in the year after chemoprophylaxis was stopped. No increase in mortality was observed but the trial was only large enough to have detected a substantial effect [8]. In Tanzania, administration of chemoprophylaxis during the first year of life led to a significant increase in the risk of malaria and anaemia during the second year of life [9]. By the age of five years the cumulative incidence of clinical attacks of malaria was similar in the intervention and control groups [10].

One of the rationales underlying the development of IPT as a malaria control tool is that by allowing some infections to occur between drug administrations, it is less likely than chemoprophylaxis to interfere with the development of naturally acquired immunity to malaria. Therefore, a number of studies have examined the malaria experience of infants in the year after they had received IPT (IPTi). In the initial study of IPTi undertaken in Tanzania [11], the investigators found that, protection appeared to persist into the second year of life with a 35% reduction in the incidence of clinical malaria in children who had previously received IPTi. However, a sustained protective effect has not been seen in subsequent studies. A suggestion of an increased risk of high density infections and of anaemia was found in two studies undertaken in Ghana [12], [13]. A meta analysis of all IPTi trials did not find clear evidence of an increase in the incidence of clinical attacks of malaria or of anaemia in the year following the intervention [1].

Only limited information is available as to whether administration of IPT to older children is associated with an increased risk of malaria in the period following the intervention. No overall increase in risk was observed in Senegal and Mali [2], [3] with incidence rate ratios (IRR) of 0.98 (95%CI: 0.82–1.17) and 1.07 (95%CI: 0.90–1.27) respectively. No significant overall increase (IRR = 1.38; 95%CI: 0.98–2.16; p = 0.059) in the subsequent malaria transmission season was observed in Ghana in children who received AS+AQ monthly [4]. A meta-analysis of available data on IPT in children found only weak evidence (IRR = 1.11; 95%CI 0.99–1.24; P = 0.07) of an increase in the incidence of malaria in the year post intervention [14]. Because of the paucity of information on the longer term impact of IPTc, we studied the subsequent malaria experience of Burkinabe children who received IPTc for one transmission season.

## Methods

The original protocol for this trial (Protocol S1), the amended protocol (Protocol S2) and supporting CONSORT checklist (Checklist S1) are available as supporting information.

Ethical approval to conduct the trial was obtained from the health ethics committee of Burkina Faso and from the London School of Hygiene and Tropical Medicine ethics committee (Ethics S1). Meetings were held with local health authorities and the communities to explain the objectives and methods of the study. Written informed consent was obtained from caregivers of children before enrolment into the trial and an independent Data Safety and Monitoring Board (DSMB) monitored the trial.

### Study area and population

The study was conducted from August 2008 to November 2009 in Boussé health district, in central Burkina Faso. Four villages (Laye, Niou, Sao and Toeghin) which each have a health centre were included. Malaria transmission in the study area is high and seasonal with most of the transmission occurring between July and October. Children aged 3–59 months residing in the study villages were enrolled. Details of the study area have been published previously [5].

### Study design

An individually randomised, double-blind, placebo-controlled trial of intermittent preventive treatment of malaria was conducted during the malaria transmission season of 2008; 3014 children were enrolled after screening. All children were given a long lasting insecticide treated net (LLIN) (PermaNet®; www.vestergaard-frandsen.com); 1509 children were randomly assigned to receive IPT with sulphadoxine/pyrimethamine (SP) plus amodiaquine (AQ) and 1505 were randomised to receive placebos. SP was given as single dose of 25 mg per kg of sulphadoxine plus 1.25 mg of pyrimethamine and AQ was given for 3 days as a daily dose of 10 mg per kg. Three rounds of IPTc were given starting in August 2008 with a one-month interval between treatments. Details of the eligibility, randomisation and intervention procedures have been presented elsewhere [5]. The intervention ended after the third course of IPT but children were followed up to the end of the subsequent malaria transmission season. The main outcome measure of the study reported in this paper is the incidence of malaria in the malaria transmission season after cessation of the intervention (2009).

### Surveillance of malaria episodes

Passive surveillance for malaria episodes was set up from the day of administration of the first dose of IPT in July 2008 until the end of November 2009. Briefly, caregivers were advised to bring their children to the village health centre at anytime if they were sick. Clinical examination was performed by a nurse. Caregivers were asked if the child had had fever in the previous 24 hours, axillary temperature was measured and signs and symptoms of illness were recorded. A rapid malaria diagnosis test (RDT) was performed and thick and thin blood films were prepared if a child had fever or a history of fever.

Children with a positive RDT who were thought to have uncomplicated malaria were treated with artesunate plus amodiaquine (AS+AQ) or artemether lumethantrine (Coartem®) at the health centre. If a child had signs or symptoms of severe malaria, treatment with quinine was provided and, when required, the child was referred to the district hospital in keeping with local guidelines. Children with an illness other than malaria were given appropriate treatment free of charge. Hospital admissions due to malaria and other causes were recorded and children's condition was monitored until release from the health centre.

### Monitoring of malaria infection, anaemia and nutrition

From July 2009 to November 2009, weekly home visits were made to monitor the prevalence of malaria infection. Each week, 150 children per treatment arm were randomly selected for a home visit. A history of fever in the previous 24 hours was recorded, axillary temperature was measured and thin and thick blood films were prepared for all children. During home visits, caregivers of children were asked if the LLIN allocated to the child was still in the house and whether the child had slept under the net the previous night.

At the end of the 2009 malaria transmission season, we performed a cross-sectional survey of all children from the original trial who were available. Children had a clinical examination performed by study medical staff. Caregivers were asked if their child had fever within the previous 24 hours and axillary temperature was measured. Thick and thin blood films and filter paper blood spots were prepared and haemoglobin concentration was measured. Children's weight and height were recorded.

During these surveys, a rapid malaria diagnostic test (RDT) was performed on the spot if a child had fever or a history of fever. If the RDT test was positive and the child was suspected of having uncomplicated malaria, s/he was referred to health centre for appropriate treatment with AS+AQ or Coartem®. Children with severe malaria or an illness other than malaria were treated as described in the previous section. Details of the procedures used during these surveys have been presented previously [5].

### Laboratory methods

Thick and thin blood films were air-dried and stained with 5% Giemsa and read independently by two laboratory technicians. The parasite count was measured against 200 white blood cells (WBC) and converted to a parasite density per µl assuming that 1 µL of blood contains 8000 WBC per µL. In the event of a discrepancy between the two readers, the slide was re-examined by a third laboratory technician. The arithmetic mean of the 2 readings was used as the final parasite density if there was agreement between two of readers. If there was no agreement after the third reading, the arithmetic mean of the three parasite densities was used.

Haemoglobin concentration was determined using a Hemocue® 321 (Hemocue AB, Angelholm, Sweden) as described previously [5].

### Sample size and power

The sample size for the first phase of the study was set to detect a 20% reduction in the incidence of clinical malaria and a 50% reduction in the incidence of admissions to hospital for severe malaria in children who had received intermittent preventive treatment. Details regarding sample size calculation for the main study have been published elsewhere (Konaté et al., 2010). Based on the requirements set out above, at least 1500 children were needed per treatment arm for the study to have a 90% power to detect reductions in the endpoint mentioned above. Thus, 1509 and 1505 children were enrolled and randomized to receive SP+AQ or placebos respectively. It was anticipated that there would be a 10%–20% of loss to follow-up in the second year. Therefore, it was estimated that with this sample size, the study would have greater than 90% power to detect a 20% increase in the incidence of clinical malaria and greater than 90% power to detect a 20% increase in parasite prevalence in subsequent high malaria transmission season post intervention in children who received IPT with SP+AQ.

### Data handling and analysis

Data entry was performed by two independent data clerks using Microsoft ACCESS and analyses were performed using STATA version 11 (www.stata.com). The intervention period was defined as the period from the first dose of IPT administration to the end of November 2008 (42 days after the third dose of IPT). The post intervention high malaria transmission period was defined as the period from July to November 2009 and the whole post intervention period was defined as the period from December 2008 to November 2009, which included the low malaria transmission season dry season (December 2008 to June 2009). Children were grouped in two age categories for adjusted analyses: children aged 3 to 23 months and children aged 24 to 59 months at the start of the intervention in August 2008.

Details of statistical procedures used in the study have been published previously [5]. Briefly, an episode of clinical malaria was defined as fever or a history of fever in the previous 24 hours together with the presence of at least 5000 asexual forms of *P. falciparum* per µl and the absence of any other obvious cause of fever. WHO's standard definition was used for severe malaria [15]. A child was not considered at risk for 21 days if he/she had experienced an episode of malaria and had been treated with an anti-malarial. The incidence of malaria was estimated as the number of malaria episodes divided by a child's time at risk. Incidence rates of malaria in children who had received IPTc or placebos during the intervention period were compared using Cox regression models. Incidence rate ratios (IRRs) were adjusted for age (used as categorical variable), sex and village. Confidence intervals around IRRs were calculated using robust standards errors to account for children who experienced multiple episodes.

Crude and adjusted IRRs for all-cause and malaria-specific hospital admissions were also estimated using a Cox regression model as described above. Prevalence ratios (RRs) of malaria infection, anaemia (Hb<11 g/dL) and moderately severe anaemia (Hb<8 g/dL) were estimated using a generalized linear model. Parasite density and Hb concentrations were compared using Student's t-test.

Anthropometric data were analysed as described previously [5]. Z-scores for weight-for-age (WAZ; underweight), height-for-age (HAZ; stunting) and weight-for- height (WHZ; wasting) were determined using the WHO child growth standard [16]. Wasting, stunting and underweight were defined as z-scores<-2 for the relevant indicator. A generalized linear model was fitted with age, sex and village as covariates to estimate PRs for wasting, stunting and underweight among children who had received IPTc compared with those who had received placebos the previous year.

## Results

### Characteristic of children at the start of the post intervention high transmission season

**Figure 1:**
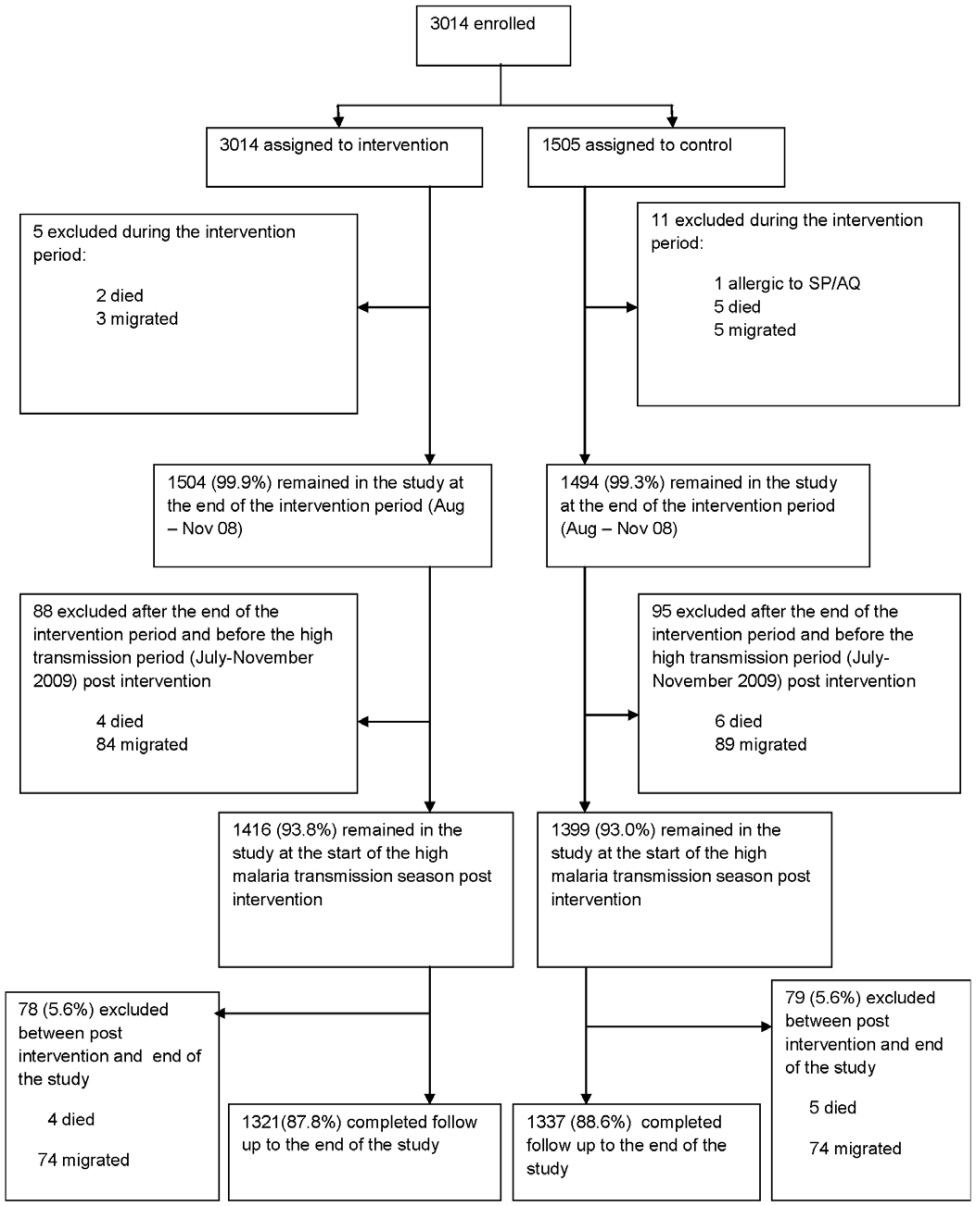
Study profile.

Baseline characteristics of the 1509 and 1505 children randomized at the beginning of the trial to receive IPTc or placebos are reported elsewhere [5]. At the beginning of the post-intervention malaria transmission season (July 2009), 1416 (93.8%) children from the former intervention arm were available for follow up compared with 1399 (93.0%) children in the former control arm (Figure 1). Six of the 93 (6.2%) children from the former intervention arm who were not available for follow up had died and 87 had migrated out of the study area. The comparable figures for the 106 children (7.0%) who had previously received placebos were 11 and 94 respectively; one child who was allergic to SP+AQ was excluded from the follow-up study. The mean age of children at the start of post-intervention follow up period was 45.9 months (95%CI 45.3–46.5). The age and sex of children in the two study arms were similar (Table 1) as was the distribution of children by village. The proportions of children who were reported to have slept under a LLIN during weekly home visits were similar between children who had received IPTc previously and children who had received placebos (93.1% versus 93.3%). These proportions of LLIN users are similar to those recorded during the intervention period.

**Table 1:** Distribution of children by age, sex and village at the beginning of the post intervention malaria transmission season (July 2009).

	Intervention	Control
	N = 1416% (n)	N = 1399% (n)
***Age (months)*** *		
6–11	15.1 (214)	17.3 (242)
12–23	22.8 (323)	21.3 (298)
24–35	20.5 (290)	20.4 (285)
36–47	22.9 (324)	20.4 (286)
> = 48	18.7 (265)	20.6 (288)
***Sex***		
Male	52.8 (748)	50.9 (712)
Female	47.2 (668)	49.1 (687)
***Villages***		
Toeghin	25.7 (364)	25.6 (357)
Niou	22.8 (323)	24.2 (339)
Laye	25.7 (364)	25.0 (350)
Sao	25.8 (365)	25.2 (353)

*Age at the time of enrolment (beginning of the trial in August 2008).

### The effect of IPTc on the incidence of clinical malaria in the post-intervention malaria transmission season

During the 2009 malaria transmission season (July 2009 to November 2009), 3493 episodes of malaria were recorded in study children; 1834 in children who had received IPTc in 2008 and 1659 episodes in those who had received placebos (table 2). Incidence rates of malaria during this period of high malaria transmission were 3.84 (95%CI; 3.67–4.02) in children who had previously received IPTc and 3.45 (95%CI; 3.29–3.62) episodes in those who had previously received placebos indicating, a small increase in the incidence of clinical malaria in the former intervention arm compared to the former control arm (IRR = 1.12; 95%CI 1.04–1.20) (P = 0.003). A similar increase in the incidence of malaria was observed when the analysis covered the whole post intervention period December 2008 to November 2009, including the 2009 dry season, (IRR = 1.12; (95%CI 1.04–1.20) (P = 0.002) (Table 3). The IRR for clinical malaria during the post intervention high transmission season (July–November 2009) was 1.16 (95%CI 1.05–1.28) (P = 0.004) for children who were aged 24 months or more at beginning of the intervention and 1.09 (95%CI 0.97–1.21) in children aged less 24 months at this time. However, there was not strong evidence that the effect of IPT during the post-intervention varied with age (P = 0.15). The mean age of children who experienced a malaria episode during the post intervention malaria transmission season did not differ between children who had previously received IPTc and those who had previously received placebos (39.2 months; 95%CI 38.5–39.6 versus 38.5 months; 95%CI 37.6–39.3 respectively) (P = 0.18).

**Table 2:** Effect of IPcT on the incidence of malaria during the post intervention malaria transmission season by age group.

	Former Intervention (SP+AQ)		Former Control					
	Episodes (child years)	Incidence rate (95%CI)	Episodes (child years)	Incidence rate (95%CI)	Unadjusted IRR (95% CI)	P-value	Adjusted*IRR (95% CI)	
**Age (months)** $								
<24	814 (173.1)	4.70 (4.39–5.03)	781 (118.0)	4.38 (4.09–4.71)	1.07 (0.95–1.19)	0.22	1.09 (0.97–1.21)	0.12
≥24+	1020 (304.3)	3.35 (3.15–3.56)	878 (302.5)	2.90 (2.72–3.10)	1.17 (1.05–.29)	0.003	1.16 (1.05–1.28)	0.004
Overall	1834 (477.5)	3.84 (3.67–.02)	1659 (480.7)	3.45 (3.29–3.62)	1.12 (1.04–1.21)	0.004	1.12 (1.04–1.20)	0.003

$ Age at the time of enrolment.

*Incidence rate ratios were adjusted for age sex and village using Cox Regression model. Confidence intervals were constructed using robust standard errors to account lack of independence of malaria episodes in children who experience multiple episodes.

**Table 3:** Effect of IPTc on the incidence of malaria during the whole post intervention period (including the dry and rainy seasons) by age group.

	Former Intervention (SP+AQ)		Former Control					
	Episodes (child years)	Incidence rate (95%CI)	Episodes (child years)	Incidence rate (95%CI)	Unadjusted IRR (95% CI)	P-value	Adjusted*IRR (95% CI)	
Whole post intervention period (December 2008–November 2009)								
Age (months)**								
<24	849 (497.0)	1.71 (1.60–1.82)	814 (500.1)	1.61 (1.52–1.74)	1.07 (0.96–1.19)	0.24	1.09 (0.97–1.21)	0.13
≥24	1106 (822.9)	1.34 (1.26–1.42)	945 (813.2)	1.16 (1.09–.24)	1.17 (1.06–.29)	0..002	1.17 (1.06–1.29)	0.002
Overall	1955 (1320)	1.48 (1.42–.55)	1759 (1312)	1.33 (1.28–1.40)	1.12 (1.04–1.21)	0.003	1.12 (1.04–1.20)	0.002

$ Age at the time of enrolment.

*Incidence rate ratios were adjusted for age sex and village using Cox Regression model. Confidence intervals were constructed using robust standard errors to account lack of independence of malaria episodes in children who experience multiple episodes.

**Figure 2:**
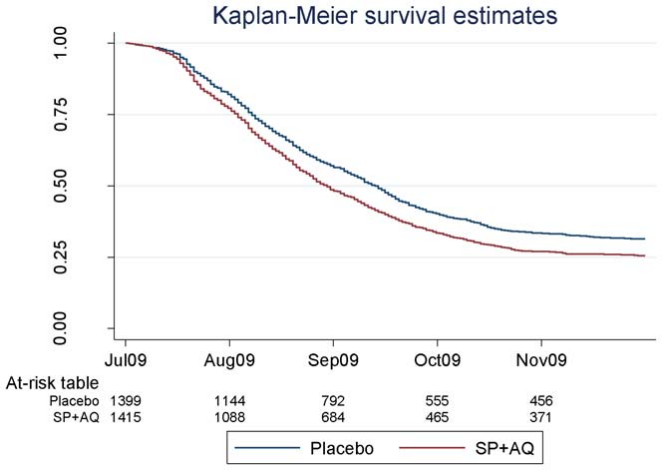
Survival time to first episode of malaria during the post intervention malaria transmission in children who had received IPTc or placebo during the previous malaria transmission season.

Kaplan-Meier survival plots (Figure 2) for time to first episode of malaria confirmed that children who had received IPTc in 2008 experienced an increased risk of clinical malaria (P<0.0001) during the subsequent malaria transmission season compared with children who had received placebos.

There was no evidence of an increased incidence of malaria post-intervention in the IPT arm when a malaria episode was defined as fever or history of fever and the presence of any parasitaemia (IRR = 1.04; 95%CI 0.97–1.10) (P = 0.29).

### Effect of IPTc on hospital admissions in the post-intervention period

From July 2009 to November 2009, 37 study children who had received IPTc the previous year were admitted to hospital, including 19 hospitalisations due to malaria compared with 39 children, 16 with malaria, among those who had previously received placebos (Table 4). There was no evidence of an increase in the incidence of all-cause hospital admissions (IRR = 0.98; 95%CI 0.61–1.56) (P = 0.93) or hospital admissions due to malaria (IRR = 1.21; 95%CI 0.61–2.43) (P = 0.58) in the children who had received IPTc previously. Similar findings were observed when data from the dry season of the post-intervention period were included in the analysis (Table 5). For the period December 2008 to November 2009, 49 and 52 all-cause hospital admissions were observed in the former intervention and control arms respectively; the adjusted IRR was 0.97 (95%CI 0.65–1.47) (P = 0.90). The numbers of hospital admissions for malaria were 19 and 16 in the two treatment arms (IRR = 1.21; 95%CI 0.61–2.43) (P = 0.58). There were 8 deaths in each treatment arm during the overall post-intervention period.

**Table 4:** Effect of IPTc on all-cause hospital admissions and hospital admissions for malaria in post intervention malaria transmission season.

	Former Intervention		Former Control					
	Episodes (child years)	Incidence rate (95%CI)	Episodes (child years)	Incidence rate (95%CI)	Unadjusted IRR (95% CI)	P-value	Adjusted*IRR (95% CI)	P-value
All-cause hospital admissions	37 (575.2)	0.064 (0.046−0.089)	39 (568.6)	0.068 (0.050–0.094)	0.94 (0.60–1.47)	0.78	0.98 (0.61–1.56)	0.93
Hospital admissions for malaria	19 (576.0)	0.033 (0.021–0.052)	16 (569.7)	0.028 (0.017–0.044)	1.17 (0.60–2.28)	0.47	1.21 (0.61–2.43)	0.58

*Incidence rate ratios were adjusted for age sex and village using Cox Regression model. Confidence intervals were constructed using robust standard errors to account lack of independence of malaria episodes in children who experience multiple episodes.

**Table 5:** Effect of IPTc on all-cause hospital admissions and hospital admissions for malaria during the whole post intervention period, including dry and rainy periods.

	Former Intervention		Former Control					
	Episodes (child years)	Incidence rate (95%CI)	Episodes (child years)	Incidence rate (95%CI)	Unadjusted IRR (95% CI)	P-value	Adjusted*IRR (95% CI)	P-value
All-cause hospital admissions	71 (1844)	0.038 (0.030–0.049)	93 (1822)	0.051 (0.042–0.062)	0.75 (0.55–1.03)	0.07	0.78 (0.55–1.09)	0.14
Hospital admissions for malaria	23 (1845)	0.012 (0.008–0.019	24 (1826)	0.013 (0.009–0.020)	0.95 (0.53–1.68)	0.86	0.98 (0.55–1.76)	0.95

*Incidence rate ratios were adjusted for age sex and village using Cox Regression model. Confidence intervals were constructed using robust standard errors to account lack of independence of malaria episodes in children who experience multiple episodes.

### Effect of IPT on malaria infection in the post-intervention period

Weekly home visits were carried out from July 2009 to November 2009 to 1495 children in each treatment arm; blood films were available from 1457 children (97.4%) who had previously received IPTc and from 1442 (96.4%) children who had previously received placebos. Five hundred and forty-eight children (37.6%) in the original IPTc group and 607 (42.1%) children in the original placebo group were parasitaemic (Table 6). There was some evidence that children from the former intervention arm had a lower prevalence of malaria infection during the post-intervention malaria transmission season than control children (adjusted PR = 0.88 95%CI 0.79–0.98) (P = 0.04). However, the median parasite density among infected children was higher in children who had previously received IPTc (5767/µL; inter quartile range 1141/µL–19091/µL) than in children who had received placebos (2878/µL; inter quartile range 670/µL–11837/µL) (P<0.0001).

**Table 6:** Effect of IPTc on malaria infection, anaemia, and anthropometric indicators during the post intervention period.

	Former intervention (SP +AQ)		Former control		Unadjusted analysis		Adjusted analysis	
	% (n)	N	% (n)	N	Prevalence ratio (95%CI)	P-value	Prevalence ratio*(95% CI)	P-value
**Prevalence of malaria infection during the post intervention period (weekly surveys)**								
Proportion with parasitaemia	37.6 (548)	1457	42.1 (607)	1442	0.89 (0.82–0.98)	0.014	0.88 (0.79–0.98)	0.039
**Prevalence of malaria infection at the end of post intervention malaria transmission season**								
Proportion with parasitaemia	40.4 (535)	1324	40.1 (522)	1302	1.00 (0.92–1.10)	0.98	1.00 (0.86–1.13)	0.99
**Prevalence of anaemia at the end of the post intervention malaria transmission season**								
Anaemia (Hb<11 g/dl)	51.2 (678)	1324	48.4 (631)	1304	1.06 (0.98–1.14)	0.15	1.06 (0.95–1.18)	0.29
Moderately severe anaemia (Hb<8 g/dl)	3.7 (49)	1324	4.7 (57)	1304	0.86 (0.57–1.28)	0.38	0.85 (0.58–1.24)	0.40
**Prevalence of wasting, stunting and being underweight at the end of post intervention malaria transmission season**								
$Wasting	6.4 (64)	928	6.3 (62)	998	1.02 (0.71–1.47)	0.90	1.04 (0.72–1.51)	0.82
†Stunting	38.4 (384)	999	39.6 (391)	987	0.99 (0.82–1.19)	0.92	0.95 (0.79–1.14)	0.92
‡Underweight	21.4 (214)	999	21.5 (212)	987	0.99 (0.80–1.23)	0.97	0.84 (0.60–1.17)	0.30

*Prevalence ratios adjusted for age, sex and village using a generalized linear model (GLM).

$ Wasting was defined as<−2 z score weight for age.

†Stunting was define as<−2 z score of height for age.

‡Underweight was defined as<−2 z score of weight for height.

Of the children who were present at the beginning of the high malaria transmission season of 2009 (1416 in the former intervention and 1399 and the control group) 1336 children in the former intervention arm and 1321 children in the former control arm participated in the cross-sectional survey at the end of the 2009 malaria transmission season. Thick and thin blood films were available from 1324 (99.1%) of children who had previously received IPT and from 1302 (98.6%) children who had previously received placebos. Overall, the prevalence of malaria infection increased with age with children aged 24 months or more being 1.72-fold (95%CI 1.49–2.00) (P<0.001) more likely to carry malaria infection than their younger siblings. The prevalence of malaria was 40.4% in children from the former IPTc group and 40.1% in children in the control group (Table 6) (adjusted PR = 1.00 95%CI 0.86–1.13) (P = 0.99). As observed during weekly home visits, the median parasite density was higher in children who had received IPTc in 2008 than in control children (3059/µL; inter quartile range 773/µL–9163/µL and 2026/µL; inter quartile range 590/µL–6640/µL and respectively) (P = 0.006).

### Effect of IPT on anaemia in the post intervention period

Hemoglobin concentration was measured at the end of surveillance in 1324 children who had previously received IPTc and in 1304 children who had received the placebos. There was no evidence that mean Hb concentration differed between children from the former intervention and control arms (10.79 g/dL; 95%CI: 10.71 g/dL–10.87 g/dL versus 10.83 g/dL; 95%CI 10.74 g/dL–10.90 g/dL) (P = 0.54) The prevalences of anaemia (Hb<11 g/dL) and moderately severe anaemia (Hb<8 g/dLl) were similar in children in the two study arms 678 (51.2%) compared with 631 (48.4%) and 49 (3.7%) compared with 57 (4.7%) respectively (RR: 1.06 95%CI: 0.95–1.18) (P = 0.29) and (RR 0.85; 95%CI: 0.58–1.24) (P = 0.40) (Table 6).

### Effect of IPT on anthropometric indicators in post-intervention period

Weight and height were measured at the end of the post-intervention period in 1336 and 1321 children in former intervention and control arms respectively; z scores for weight for age, height for age and weight for height were obtained from 999 children in former intervention arm and from 987 children in the former control arm (Table 6). The prevalence of wasting was 6.4% in children in the previous IPTc group (64) and 6.3% in the control children (62). The proportion of children in the previous IPTc and control arms who were stunted were 38.4% (384) and 39.6% (391) respectively and the proportions of children who were underweight children were 21.4% (214) and 21.5% (212) respectively. There was no evidence of an increased in risk of wasting (P = 0.82), stunting (P = 0.92) or being underweight (P = 0.30) in children who had previously received IPTc (Table 6).

## Discussion

Children previously enrolled in a trial of IPTc with SP+AQ were followed up for a further 12 months to investigate whether children who had received IPTc were at increased risk of malaria (rebound malaria) in the subsequent malaria transmission season as a consequence of impaired development of acquired immunity due to reduced exposure to malaria. The study found evidence that children who had received IPTc during the previous year experienced a small increase in the incidence of clinical malaria in the subsequent malaria transmission but there was no shift in the age at which these children had malaria. In a parallel study in Mali, which used very similar methods, a similar small increase (9%; 95%CI: −1–21) was observed [17]. A small, and statistically non significant, increase (IRR = 1.07; 95%CI: 0.90–1.27) had been reported from an earlier study in children who received SP bimonthly in Kambila, Mali [3]. Another IPT study in children, which tested 3 different drug regimens (SP bimonthly, AS+AQ bimonthly and AS+AQ monthly) in Ghana, reported an excess of malaria in all treatment groups in the year after intervention was stopped, but the differences between intervention and placebo groups were not statistically significant [4].

Previous IPT studies that examined whether IPT was followed by a ‘rebound’ in malaria were conducted in areas with relatively low use of ITNs and low malaria transmission intensity. In our study area malaria transmission is now moderate and 93% of the children slept under an ITN. This could also interfere with the development of acquired immunity to malaria [7] although no direct evidence to support this contention has been found [18]. ITN coverage and malaria transmission intensity differed between sites. In a forest region of Ghana, where malaria transmission intensity is higher than in the study area in Burkina Faso, no evidence of rebound malaria morbidity in children was observed when IPT with AS+AQ was combined with home management of malaria [19], a strategy that combines prevention and treatment. However, there are currently insufficient data to indicate whether there is an association between levels of malaria transmission and rebound morbidity.

The effect of IPTc on clinical malaria during the post intervention year appeared slightly higher in older children who were likely to have been more exposed to malaria infection in their lifetime than in their younger siblings. This observation is consistent with previous reports from Senegal [2] which noted a greater relative increase in the incidence of malaria in the year post intervention in older children who had received AS+SP. It is worth noting that the number of children who still aged less than 24 months in our study was relatively small in the post intervention year; the study was only powered to detect a 20% excess in clinical malaria in children (younger and older children combined). In addition, a test for interaction found no evidence that the effect of IPTc on clinical malaria during the post intervention year varied with age. In contrast, a significantly higher increase in the incidence of malaria was observed in younger children (3–11 months at enrolment) than in older children (12–59 months) who received AS+AQ monthly in Ghana [4]. Our study did not demonstrate persisting protection in younger children during the high malaria transmission period post intervention as was the case in Senegal [2].

The small excess of clinical malaria in the post intervention period in children in the former intervention arm was not accompanied by an increase in all-cause hospital admissions or hospital admissions due to malaria. However, our study was not powered to detect a rebound effect in hospital admission, and caution is required in the interpretation of these results.

Children who had received IPTc during the previous year were not at increased risk of harbouring a malaria infection at the end of the subsequent malaria transmission season nor did they have a higher prevalence of malaria infection during weekly surveys, despite their slightly increased susceptibility to clinical malaria. Although children who had received IPT in 2008 appeared to have lower prevalence of malaria infection in the subsequent malaria transmission season than control children, they had a reduced ability to control parasite density which is important for the development of clinical malaria, and may partially explain the slight increase in clinical malaria. Similar findings have been observed previously in children [2], [4] and infants [11] who have received IPT. High parasitaemia is an important risk factor for malaria anaemia. However, in this study, children who had received IPTc were not at an increased risk of anaemia or moderately severe anemia at the end of the subsequent malaria transmission season. This finding is consistent with previous reports on from Senegal [2] and Ghana [4], [19].

A limitation of this study, and previous studies that examined the role of IPTc in causing rebound malaria morbidity, is that the intervention was given during only one malaria transmission season. This may not be sufficient to significantly impair the acquisition of immunity and induce a large increase in malaria morbidity. In addition, most of these children enrolled in the studies undertaken so far had experienced significant exposure to malaria infection prior to the intervention and may, therefore, have developed some level of immunity to malaria before receiving IPTc which may have been sufficient to counterbalance the potential negative effects of reduced exposure to malaria on malaria morbidity in the subsequent transmission season. If IPTc is to be deployed on a major scale and given annually for the first few years of a child's life, it will be essential that the impact of several years of IPTc on the development of immunity to malaria is investigated carefully.

## Supporting Information

Protocol S1 Trial protocol
(DOC)

Protocol S2 Protocol amendment
(DOC)

Checklist S1 Consort Checklist
(DOC)

Ethics S1 (PDF)
