# Supplementary material for: Morbidity from Malaria in Children in the Year after They Had Received Intermittent Preventive Treatment of Malaria: A Randomised Trial
Source: PLoS One. 2011 Aug 12;6(8):e23391. doi: 10.1371/journal.pone.0023391 (PMC3155539; doi:10.1371/journal.pone.0023391)
Supplement: Ethics S1 — (PDF) [file pone.0023391.s004.pdf]

LONDON SCHOOL OF HYGIENE
& TROPICAL MEDICINE

ETHICS COMMITTEE

APPROVAL FORM
Application number: **5275**

Name of Principal Investigator **Professor Brian Greenwood**

Department **Infectious and Tropical Diseases**

Head of Department **Professor Simon Croft**

Title: **A trial of the combined impact of intermittent preventive treatment and insecticide treated bednets on morbidity from malaria in African children.**

This application has been approved by the Committee.

Chair .... T. W. Meade ....
Professor Tom Meade

Date ....… ….19 March 2008..………………………………

Approval is dependent on local ethical approval having been received.

Any subsequent changes to the consent form must be re-submitted to the Committee.
